# DEC1 promotes progression of *Helicobacter pylori*‐positive gastric cancer by regulating Akt/NF‐κB pathway

**DOI:** 10.1111/jcmm.17219

**Published:** 2022-02-04

**Authors:** Yanfei Jia, Yanyan Liu, Jingyu Zhu, Liang Liu, Xiaoli Ma, Duanrui Liu, Shuyi Han, Lulu Zhang, Zhi‐Qiang Ling, Yunshan Wang

**Affiliations:** ^1^ Research Center of Basic Medicine Jinan Central Hospital Shandong First Medical University Jinan China; ^2^ Research Center of Basic Medicine Jinan Central Hospital Cheeloo College of Medicine Shandong University Jinan China; ^3^ Department of Clinical Laboratory Qilu Hospital Shandong University Jinan China; ^4^ Department of Gastroenterology Jinan Central Hospital Shandong First Medical University Jinan China; ^5^ Medical Research & Laboratory Diagnostic Center Jinan Central Hospital Shandong First Medical University Jinan China; ^6^ Zhejiang Cancer Institute The Cancer Hospital of the University of Chinese Academy of Sciences (Zhejiang Cancer Hospital) Institute of Basic Medicine and Cancer (IBMC) Chinese Academy of Sciences Hangzhou China

**Keywords:** differentiated embryo‐chondrocyte expressed gene 1, gastric cancer, *H. pylori*, progression

## Abstract

*Helicobacter pylori (H*. *pylori)* infection plays a crucial role in the initiation and progression of gastric cancer (GC). Differentiated embryo‐chondrocyte expressed gene 1 (DEC1) is dysregulated in some cancers and may regulate cell proliferation in specific contexts. Of note, DEC1 is emerging as one of the important factors regulating cellular responses in microenvironment. However, the triggers and precise regulation mechanism for DEC1 during inflammatory carcinoma transformation of GC are unclear. In this study, we identified DEC1 was upregulated in both *H*. *pylori*‐infected gastric tissues and GC cells. DEC1 expression was positively associated with *H*. *pylori* infection status and GC progression. DEC1‐positive expression indicated a poorer prognosis in *H*. *pylori*‐positive GC. DEC1 was required for *H*. *pylori*‐induced GC cells proliferation. Mechanistically, *H*. *pylori* infection significantly activated Akt/NF‐κB signal pathway and this induction depend on DEC1 expression level in GC cells. Importantly, their interaction pathway was further verified by *H*. *pylori*‐positive gastritis mice model. Taken together, our findings identified a novel function of DEC1 in GC. *H*. *pylori* infection induce DEC1 expression, and which leading to the progression of GC through activating Akt/ NF‐κB signalling pathway. Blocking DEC1/Akt/NF‐κB, therefore, presents a promising novel therapeutic strategy for *H*. *pylori*‐positive GC.

## 1 INTRODUCTION

Gastric cancer (GC)is a complex disease, and its pathogenesis involves a variety of signal transduction pathways. Most patients with GC have no obvious symptoms in the early stage, but have entered the advanced stage at the time of diagnosis. As the only definite cause of gastric adenocarcinoma, *Helicobacter pylori (H*. *pylori)* has been classified as type I carcinogen by the International Agency for Research on Cancer (IARC) in 1994,^1^ with almost 90% of new cases of noncardia GC attributed to this bacterium. However, more than half of the world's population is infected with *H*. *pylori*, only 2– 3% of infected people develop GC.^2^, ^3^ Studies have pointed out that *H*. *pylori* infection could disturb the dynamic balance between cell proliferation and apoptosis and promote GC carcinogenesis.^4^, ^5^, ^6^ Therefore, understanding the complexity of tumorigenic interactions between *H*. *pylori* and GC cells can provide a theoretical basis for the treatment of *H*. *pylori*‐positive GC.

Accumulating evidence has demonstrated that differentiated embryo‐chondrocyte expressed gene 1 (DEC1) is one of the important factors regulating cellular responses in microenvironment. DEC1 is a hypoxia‐induced gene involved in cell differentiation, proliferation and apoptosis.^7^ DEC1 could resist oxidative stress‐mediated cell death in skeletal muscle and podocytes.^8^, ^9^ Chemotherapy drugs, tumour necrosis factor (TNF)‐α and transforming growth factor (TGF)‐β could induce DEC1 expression and regulate apoptosis‐related signalling pathways.^10^, ^11^, ^12^ DEC1 also can drive inflammation and enable typical immune responses in autoimmunity and infection.^9^, ^10^ DEC1 is a key regulator of cytokine production by human T cells and essential for mitochondrial metabolism in tumour infiltrating lymphocytes.^13^, ^14^, ^15^, ^16^, ^17^ *H*. *pylori* infection is an intrinsic niche for tumorigenesis and GC progression. However, it is not clear whether the inflammatory environment regulates the expression of DEC1 in cancer cells. Furthermore, in cancer‐related studies, the role of DEC1 as a promoter or inhibitor of cancer remains controversial.^3^, ^12^, ^18^, ^19^, ^20^, ^21^, ^22^, ^23^, ^24^ DEC1 expression level also can be used to predict the effect and clinical prognosis of cancer after radiotherapy and chemotherapy.^6^, ^20^, ^25^ Our previous studies have shown DEC1 is highly expressed in GC, and it is negatively correlated with the degree of differentiation of tumours.^19^, ^26^ However, the original causes that initiate the increased expression of DEC1 in GC during carcinogenesis are still unknown.

It is well established that some oncogenic signalling pathways, including the PI3 kinase‐Akt, β‐catenin‐WNT, Hedgehog/GLI, ERK, JNK and NF‐κB pathways, were involved in *H*. *pylori* pathogenesis.^27^, ^28^, ^29^, ^30^, ^31^ Therefore, there are some key genes could determine the pathogenic effect of *H*. *pylori* by regulating these oncogenic signalling pathways. It has been shown DEC1 is upstream of p‐Akt, p‐GSK3β, p53, caspase‐8 and Fas.^11^, ^32^, ^33^ Investigation the complex intracellular signalling events related to the role of DEC1 in *H*. *pylori*‐infected GC may be important for further elucidation of its role in the pathogenesis of tumours.

In this study, we find that *H*. *pylori* induce DEC1 expression in GC. Moreover, we validate DEC1 is required for the oncogenic role of *H*. *pylori* by activation p‐Akt/ NF‐κB signal pathway. The role of DEC1 in the progression of *H*. *pylori*‐positive GC may imply DEC1 as a potential therapeutic target.

## 2 MATERIAL AND METHODS

### 2.1 Bioinformatics analysis

GSE chips were downloaded from the GEO website (https://www.ncbi.nlm.nih.gov/ geo/), and gene expression data of each sample in the chips were normalized using R 3.3.3 software. Bioconductor limma package was used to analyse the differential expression genes. The criteria for differentially expressed genes were |logFC|>1.5 and *p* Value<0.05. Gene expression data files were extracted by R 3.3.3 software. Differential gene expression analysis was performed using the edgeR package and DESeq package. To annotate the different underlying biological processes of dysregulated DEC1 in the *H*. *pylori*‐positive GC, Gene Set Enrichment Analysis (GSEA) was performed using GSEA 2.0 software. The mRNA expression data of 55 *H*. *pylori*‐positive GC patients were downloaded from GEO (GSE66254) database. The DEC1 high‐expression group and DEC1 low‐expression group were set up according to the median expression value. Annotated gene sets c2.cp.kegg.v5.2. symbols.gmt was selected as the reference gene sets. The false defection rate q value <0.05 and normalized enrichment score (NSE) >1 were selected to sort the pathways enriched in each phenotype.

### 2.2 *H*. *pylori* culture

*H. pylori* standard strain NCTC 11637 was kindly provided by Dr. Jihui Jia (Department of medical microbiology, Shandong University, Shandong, China). Columbia blood agar medium containing 7% defibred sheep blood was used as culture medium. *H*. *pylori* were incubated for 2–3 days under microaerobic, 35℃ and 90% humidity conditions. The concentration of bacteria was measured by Densimat. Gram staining, oxidase test, catalase test and urease test were used for bacterial identification.

### 2.3 Cell culture

Human gastric epithelial cells GES‐1, gastric adenocarcinoma cell lines MKN‐45, HGC‐27 and MGC‐803 were purchased from IBCB (Shanghai Institute of Biochemistry and Cell Biology, Chinese Academy of Science). RPMI‐1640 medium containing 10% foetal bovine serum (FBS) (Hyclone, USA) was used for cell culture. Cells were cultured in a cell culture chamber at 5% CO2 at 37℃. 2 x 10^6^ cells were added into 6cm culture dishes. *H*. *pylori* were added into the cell culture dish (MOI = 10). Cells were harvested after co‐culture for a certain period of time. Extract total protein, total RNA or use cells for cell‐related functional experiments. Akt inhibitors were purchased from Selleck Ltd(USA) and used at a concentration of 10μM.

### 2.4 Clinical samples

Eighty human gastritis tissues were purchased from Alenabio Biotechnology Co., Ltd (Xi'an China). A total of 80 samples of primary GC and tumour adjacent gastric mucosa were collected from the department of pathology, Jinan central hospital. 32 samples of *H*. *pylori*‐positive GC were collected. The patients did not receive any chemotherapy or radiotherapy before the operation. Pathological TNM staging is based on the 8th AJCC Cancer Staging System. This study was approved by the ethics committee of Jinan central hospital.

### 2.5 Immunohistochemical staining of paraffin sections

Immunohistochemical (IHC) staining was performed by the streptavidin‐peroxidase method. In brief, after deparaffinization, hydration and antigen retrieval, the tissue sections were incubated with the primary antibodies including anti‐DEC1 (1:200; Sigma‐Aldrich, St. Louis, MO) overnight at 4°C. Then, the slides were washed with phosphate‐buffered saline (PBS) and incubated with enhanced enzyme‐labelled goat anti‐rabbit IgG polymer at room temperature for 20 minutes (1:2000; KIT‐5010; Maixin‐Bio, Fujian, China) in PBS for 30 minutes at 37°C. Subsequently, slides were visualized by incubation with a 3,3‐diaminobenzidine solution. The nucleus was counterstained with haematoxylin. Immunohistochemical analysis was carried out by two independent investigators concurrently.

### 2.6 Cell transfection and lentiviral transduction

The DEC1 vector (Shanghai GeneChem, Shanghai, China) was prepared with full‐length complementary DNA (cDNA). An empty vector GV492 (EV) was used as the control. shRNAs specific to DEC1 expression were from our previous study.^19^ The target sequence of the shDEC1 was 5’‐CATTGCCCTGCAGAGTGGTTTACAACTTCC TGTCAGATTGTAAACCACTCTGCAGGGCAATG‐3’. The lentiviral vectors were cotransfected with packaging vectors psPAX2 and pMD2G into 293T cells for lentivirus production. Transfection was performed using the Lipofectamine 2000 Transfection Reagent (Invitrogen, Carlsbad, CA) according to the manufacturer's instructions. Puromycin (2 μg/ml, Sigma) was used to select stable clones for at least 1 week. At the indicated time points, the cells were harvested for mRNA and protein analysis as well as for other assays.

### 2.7 Quantitative real‐time PCR (qRT‐PCR) analysis

Total RNA was extracted using Trizol reagent (Invitrogen). Reverse transcription reaction kit (Takara, Japan) was used for the synthesis of the cDNA. The operation is carried out according to the manufacturer's instructions. The real‐time fluorescent quantitative polymerase chain reaction was performed by ABI 7500 systems. Relative gene expression was calculated using 2(‐ΔΔCt) (Livak). Each experiment was repeated three times.

### 2.8 Western blot analysis

RIPA lysate (containing PMSF and phosphatase inhibitors) was used for the extraction of total cell protein (Beyotime Ltd, China). Total protein was quantified by bicinchoninic acid protein assay (Thermo Fisher Scientific, Rockford, IL). Proteins were separated by 10% sodium dodecyl sulphate polyacrylamide gel electrophoresis gel and transferred to polyvinylidene difluoride membranes (Millipore, Boston, MA). According to the instructions, the primary antibodies against CagA (ab90490, Abcam, Cambridge, MA, USA), p‐Akt1(2118–1, Epitomics, Cambridge, MA, USA) were diluted at a ratio of 1:1000. Primary antibodies against DEC1 (sc‐101023, Santa Cruz Biotechnology, USA) were diluted at a ratio of 1:200. Primary antibodies against NF‐κB (sc‐8008, Santa Cruz Biotechnology, USA), Bcl‐2(12789–1‐AP, Proteintech Group, China), Survivin(10508–1‐AP, Proteintech Group, China) and Bax(ab32503, Abcam, Cambridge, MA, USA) were diluted at a ratio of 1:500. Primary antibodies against GAPDH (10494–1‐AP, Proteintech Group, China) were diluted at a ratio of 1:2000. Incubate in second antibody (1:10000, ab150077 and ab150117, Abcam, Cambridge, MA, USA). Chemiluminescent detection was performed by Fluor ChemE system (Cell Biosciences, USA) using an enhanced chemiluminescent reagent.

### 2.9 Cell proliferation assay

Cell Counting Kit‐8 (Dojindo, Japan) was used to detect cell proliferation. The experiment was carried out according to the instructions. In a 96‐well plate, 5 × 10^3^ cells were added to each well. The next day, 10μl CCK‐8 reagents were added to each well and incubated with cells at 37°C for 2 hours. Use microplate reader (Bio‐Rad, USA) to measure 450nm absorption. Test once a day for 5 days. The experiment was repeated three times.

### 2.10 EdU

The operation is carried out according to the instructions of the cell‐light TM EdU Apollo 567 in vitro kit (Ribobio Ltd, China). In 96‐well plates, 5 × 10^3^ cells were added to each well. 100μl culture medium (0.1%EdU) was added to each well for 2 hours before fixation in 4% paraformaldehyde. After permeabilization with 0.5% Triton X‐100, 100 μL 1×Apollo‐staining reaction liquid was added to cells at 37°C for 30 minutes, the cells were counterstained with 4′,6‐diamidino‐2‐phenylindole(DAPI) and imaged using a fluorescence microscope (Olympus, Tokyo, Japan). Proliferating cells was fluoresce red. Nuclei were counterstained with blue‐fluorescent DNA stain DAPI.

### 2.11 Cell clone formation test

In the 24‐well plate, 1000 cells were added to each well. After 10 days of incubation in the cell incubator, 500 μl methanol was added to each well for 15 minutes. After cleaning with PBS buffer two times, each well was dyed with 0.1% crystal violet dye for 30 minutes.

### 2.12 Detection of apoptosis by flow cytometry

The operation steps are carried out according to the relevant instructions of the annexin‐V/7‐aminoactinomycin‐D (7‐AAD) kit (Becton‐Dickinson, San Diego, CA). Cells were washed twice with cold PBS buffer and suspended with 1 × Annexin V combination buffer. The cell concentration was adjusted to 1 × 10^6^ cells/ml. 5μl PE Annexin V and 5μl 7‐AAD were added to 100 5μl cell suspension for 15 minutes at room temperature in the dark. The samples were analysed by FACS Calibur System (BD, USA).

### 2.13 Animal experiments

30 male C57BL/6J mice aged 4–5 weeks were raised in a SPF animal laboratory. Divide mice into the control group and the experimental group. We randomly divided 30 mice into 2 groups which were a control group (n = 15) and the infection group (n = 15). Mice subjected by intragastric administration (IG) with PBS or H. *pylori* 11637. Repeat this infection process every other day 5 times. Three months later, the mice were sacrificed and gastric mucosae were retained. The experimental operation was examined and approved by the Ethics Committee of Shandong University.

### 2.14 Statistical analysis

All in vitro experiments were repeated more than three times, and three parallel groups were set up each time. The analysis data were expressed as (mean±SD). Use group t‐test for two independent samples. The relationship between DEC1 expression and pathological parameters was examined by Fisher's exact test. *p* < 0.05 means the difference has statistical significance.

## 3 RESULTS

### 3.1 DEC1 expression is positively correlated with *H*.*pylori* infection status and the progression of GC

**FIGURE 1:**
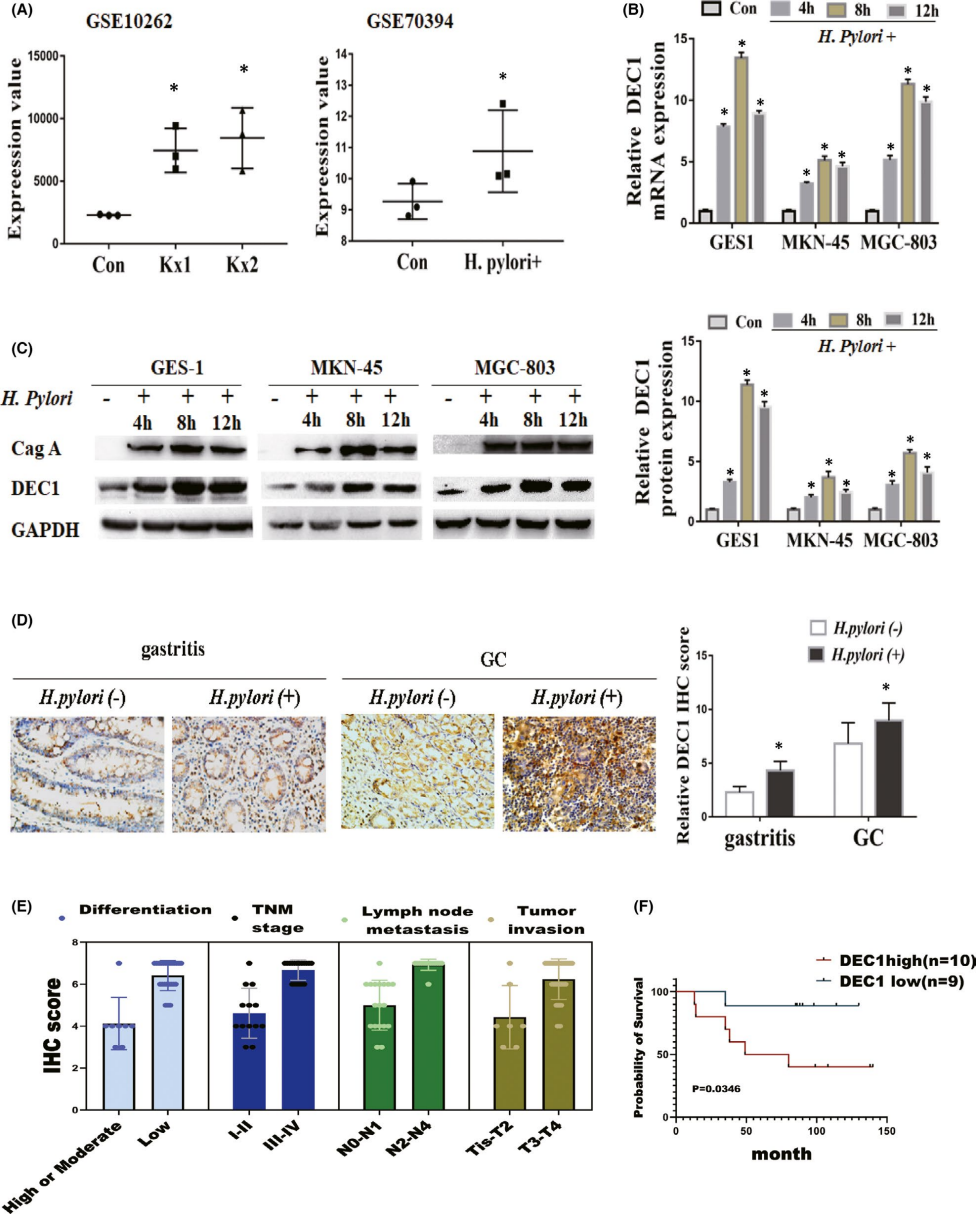
DEC1 expression is positively correlated with *H*. *pylori* infection status and the progression of GC. A. GEO RNA sequencing database analysis of the DEC1 expression in *H*. *pylori*‐infected gastric cells. The data were derived from the GSE00262 and GSE70394. B. qRT‐PCR results of cells co‐cultured with *H*. *pylori* for 4h, 8h and 12h. C. DEC1 protein expression after co‐cultured with *H*. *pylori* especially at 8h time point. D. DEC1 immunohistochemical stain of GC with or without H. Pylori. Magnification: 40×. E. Associations between DEC1 expression levels and clinicopathological characteristics of *H*. *pylori*‐positive GC. F. Survival analysis of DEC1 expression in *H*. *pylori*‐positive GC. All data are presented as mean±standard deviation of at least three independent experiments. Statistic process: t‐test, chi‐square or Fisher exact tests. *p < 0.05

In our previous study, we demonstrated that the DEC1 was upregulated in GC tissues compared to adjacent normal tissues. To dissect the potential relationship between DEC1 expression and infection of *H*. *pylori* in GC development, we firstly analysed the relative expression of DEC1 in public microarray data from the GEO database. We downloaded two publicly available mRNA chips about interaction between *H*. *pylori* and gastric cells (Figure 1A). The strains Kx1 and Kx2 used in GSE10262 chip came from a same patient. Kx1 was isolated from the gastric tissue of patients with chronic atrophic gastritis four years ago, and Kx2 was isolated from patients with gastric adenocarcinoma four years later. Differential gene expression analysis of the control group VS Kx1 infection group and control group VS Kx2 infection group showed that both Kx1 and Kx2 could upregulate DEC1 expression in non‐progenitor gastric epithelial cells. In addition, the DEC1 logFC value (1.91) of Kx2 group was higher than that of Kx1 group (1.68), which indicated that the ability of *H*. *pylori* strains to upregulate DEC1 was also improved during the long‐term interaction between H. pylori and gastric cells, and they participated in the whole process of malignant transformation from gastritis to GC. In GEO Dataset GSE70394, GC cell line AGS was incubated with *H*.*pylori* strain NCTC11637 (Cag A positive). An increased expression of DEC1 was evident at 24h post‐incubation (Figure 1A).

To verify these microarray results, GC cell lines (MKN‐45 and MGC‐803) and gastric normal mucosal epithelial cell line (GES‐1) were incubated with *H*. *pylori* strains NCTC 11637. DEC1 mRNA and protein levels were significantly increased over time with *H*. *pylori* infection in all three cell lines (Figure 1B‐C). In each cell line, the expression of DEC1 in 8 h was highest (Figure 1B‐C). So we used 8 h for further experiment.

Finally, we determined whether the results from the microarray data and cell lines have any clinical relevance. A total of 160 patients with gastric or GC were randomly enrolled and divided into *H*. *pylori*‐positive and *H*. *pylori*‐negative groups. DEC1 expression was positively associated with H. pylori infection status among these patients (Figure 1D *p* < 0.05). Combined with the pathological results after operation, 32 cases of *H*. *pylori*‐positive GC was further analysed. The results showed that the expression of DEC1 was negatively correlated with the degree of differentiation (Figure 1E) (*p* = 0.001), and positively correlated with the depth of invasion (*p* < 0.05), lymph node metastasis (*p* < 0.05) and TNM stage (*p* < 0.01). Survival curves further confirmed that survival time was lower in patients with high DEC1 levels in *H*.*pylori*‐positive GC (Figure 1F). Collectively, above results hint an important link between enhanced DEC1 expression and *H*. *pylori*‐infected status in GC.

### 3.2 DEC1 is an important mediator for *H. pylori* to perform tumorigenic activities

**FIGURE 2:**
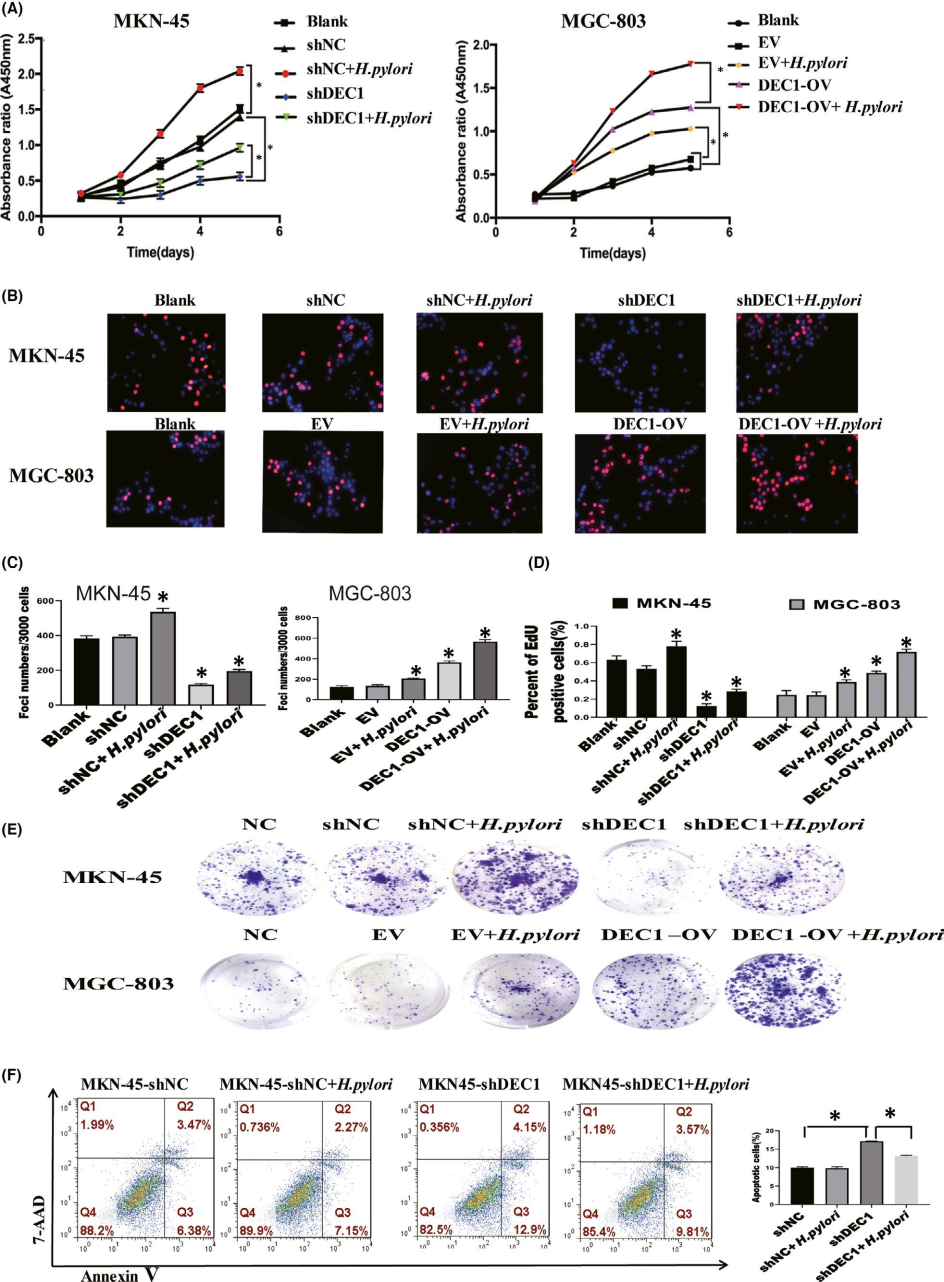
DEC1 expression increased the promotion effect of *H*. *pylori* on cell proliferation. A. The results of CCK‐8 continuous detection. B,D. EdU test and quantitative analysis. CE. Colony‐forming unit assays and quantitative analysis. F. Annexin V‐(7‐AAD) staining to assess the apoptosis rate. Each experiment was repeated for three times. EV, empty vector; OV, overexpression vector. t‐test, *p < 0.05

**FIGURE 3:**
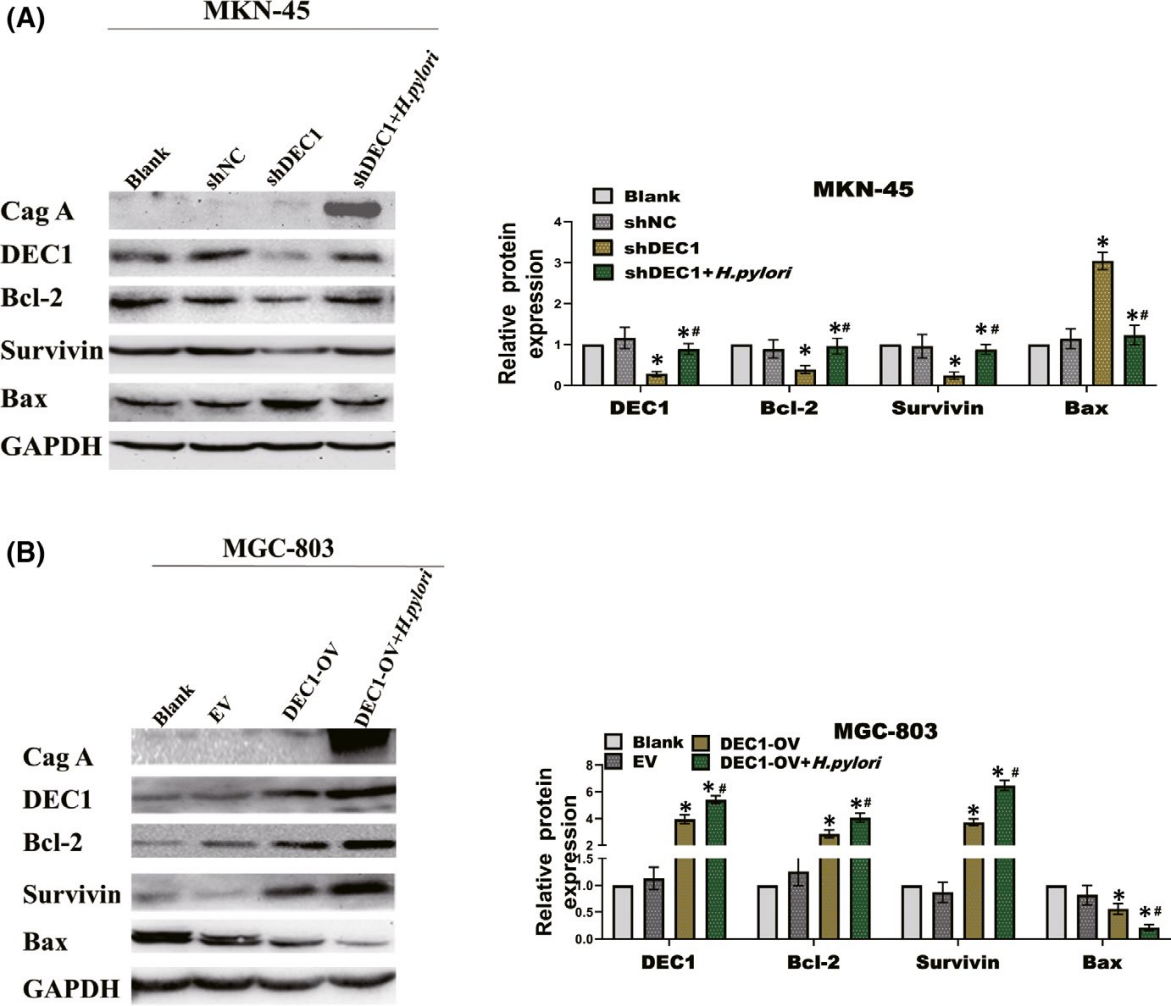
Protein expression levels of MKN‐45 and MGC‐803 after co‐cultured with *H*. *Pylori*. Western blot analysis of protein expression levels of the proliferation and apoptosis genes (Bcl‐2, Survivn and Bax) A. MKN‐45 cells. B. MGC‐803 cells. Each experiment was repeated for three times. EV, empty vector; OV, overexpression vector. *p < 0.05

Our previous study has shown the DEC1 expression was high in MKN‐45, but low in MGC‐803.^19^ We downregulated DEC1 expression in MKN‐45 cells by using shDEC1 lentivirus vector and upregulated DEC1 expression in MGC‐803 cells. CCK‐8, EdU and Colony formation assays results showed *H*. *pylori* infection increased the GC cell proliferation and the DEC1‐shRNA abrogated *H*. *pylori*‐mediated biological effects. Overexpression of DEC1 in MGC‐803 increased the promotion of cell proliferation with *H*. *pylori* infection. The apoptosis rate was assessed by flow cytometry with Annexin V‐ (7‐AAD) staining. DEC1 knockdown eliminated the inhibitory effect that *H*. *pylori* had on apoptosis (Figure 2). Protein expression levels of the proliferation and apoptosis genes (Bcl‐2, Survivin and Bax) were consistent with the cellular biological behaviour. Overexpression DEC1 in MGC‐803 had the opposite effect (Figure 3). These results indicate that *H*. *pylori* infection promotes the tumorigenesis of GC via inducing the expression of DEC1.

### 3.3 DEC1 regulates Akt/NF‐κB pathway to support *H. pylori*‐induced proliferation in GC cells

**FIGURE 4:**
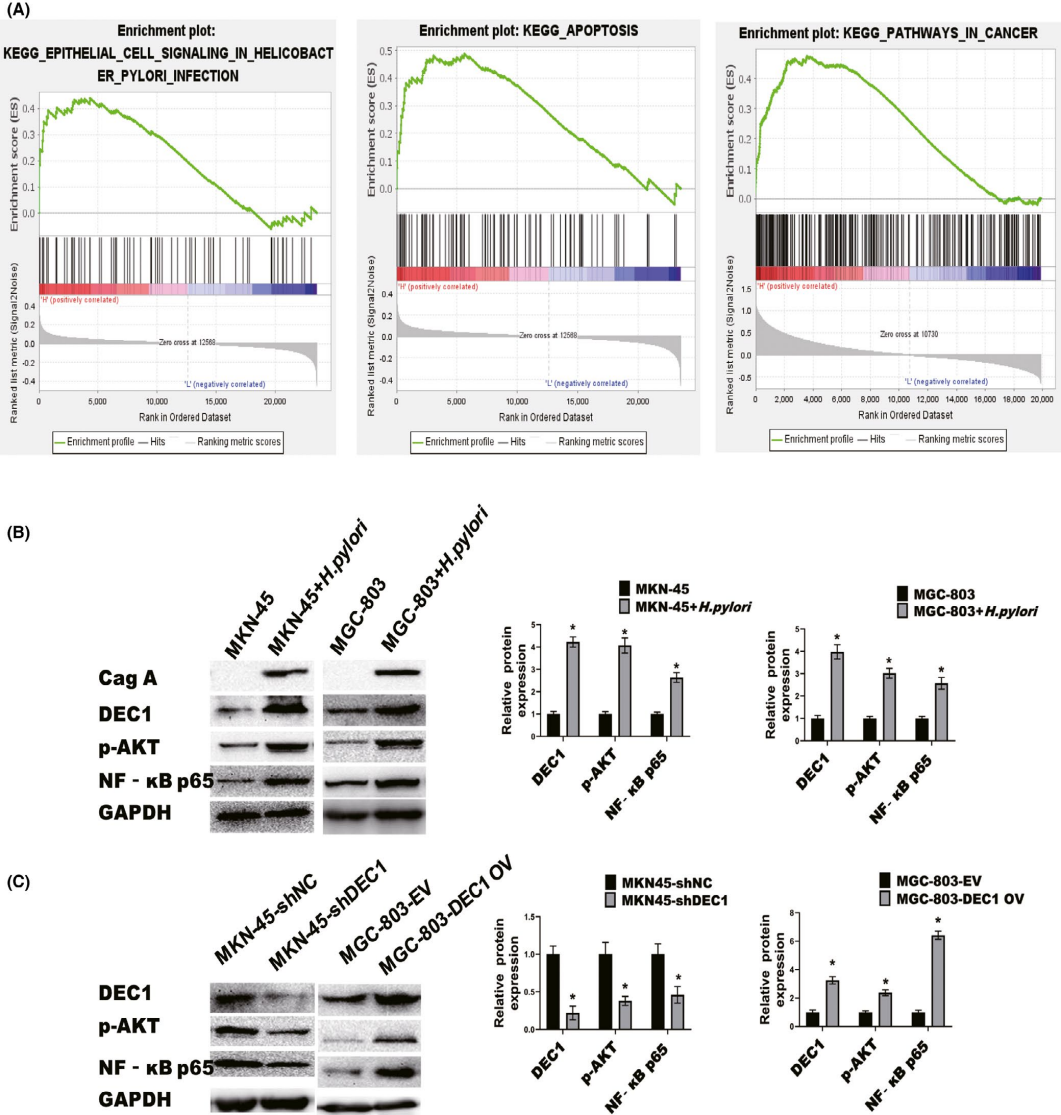
DEC1 regulates Akt/NF‐κB pathway in *H. pylori*‐infected GC cells. A. GSEA of GSE62254 data set revealed that higher DEC1 expression in *H*. *pylori*‐positive GC was significantly correlated with the epithelial cell signalling in Helicobacter pylori infecting, apoptosis and pathway in cancer. B. Effect of H. Pylori infection on the expression of p‐Akt and NF‐κB p65. (MOI = 10, 8h) C. DEC1 regulates p‐Akt and NF‐kb p65 expression in GC cells. All the experiments were repeated three times. EV, empty vector; OV, overexpression vector. *p < 0.05

**FIGURE 5:**
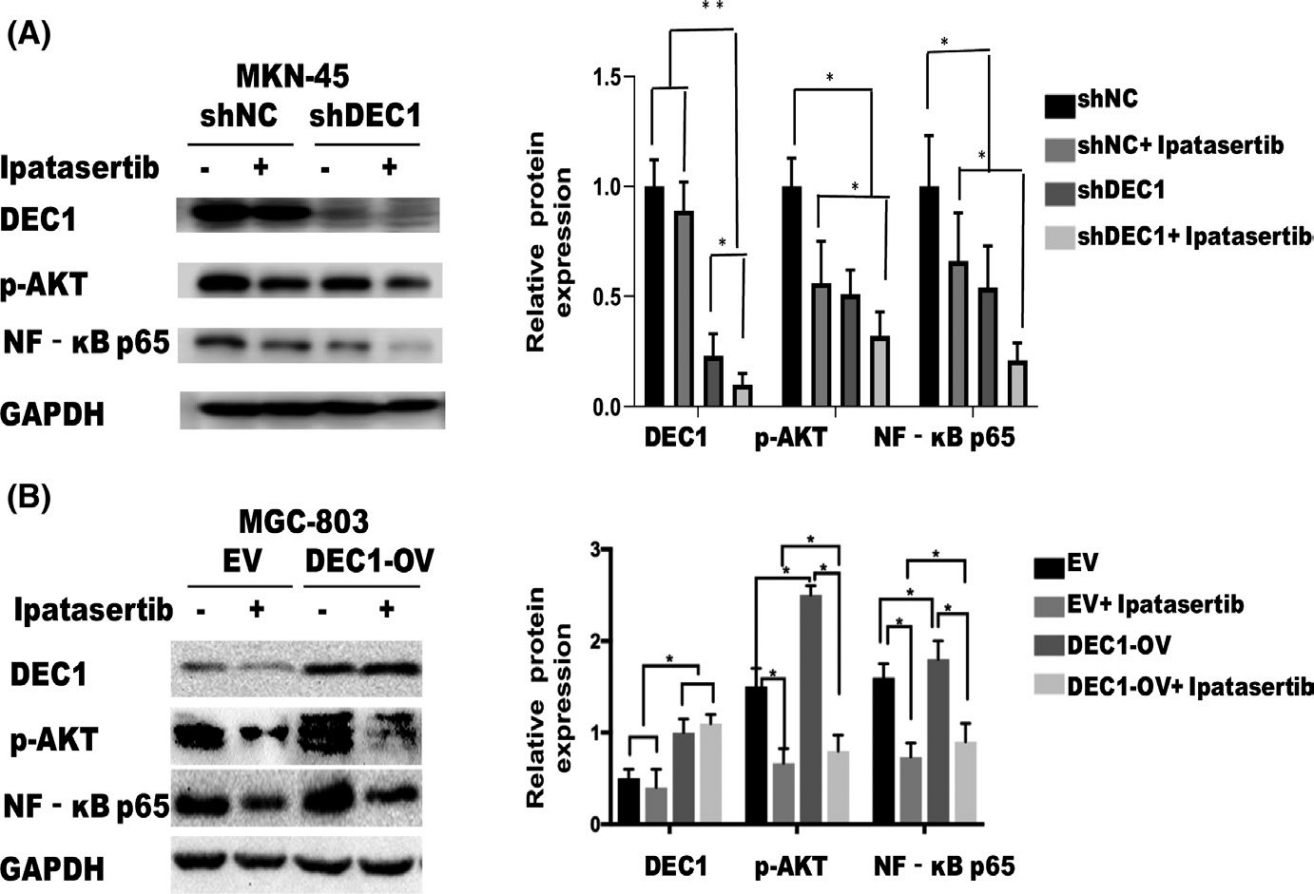
Akt inhibitor could further regulate the expression level of NF‐κB in GC cells. MKN‐45 and MGC‐803 cells were treated with ipatasertib, a specific inhibitor of Akt, and DEC1, p‐Akt and NF‐κB p65 protein expression were detected by Western blot. All the experiments were repeated three times. EV, empty vector; OV, overexpression vector. *p < 0.05

**FIGURE 6:**
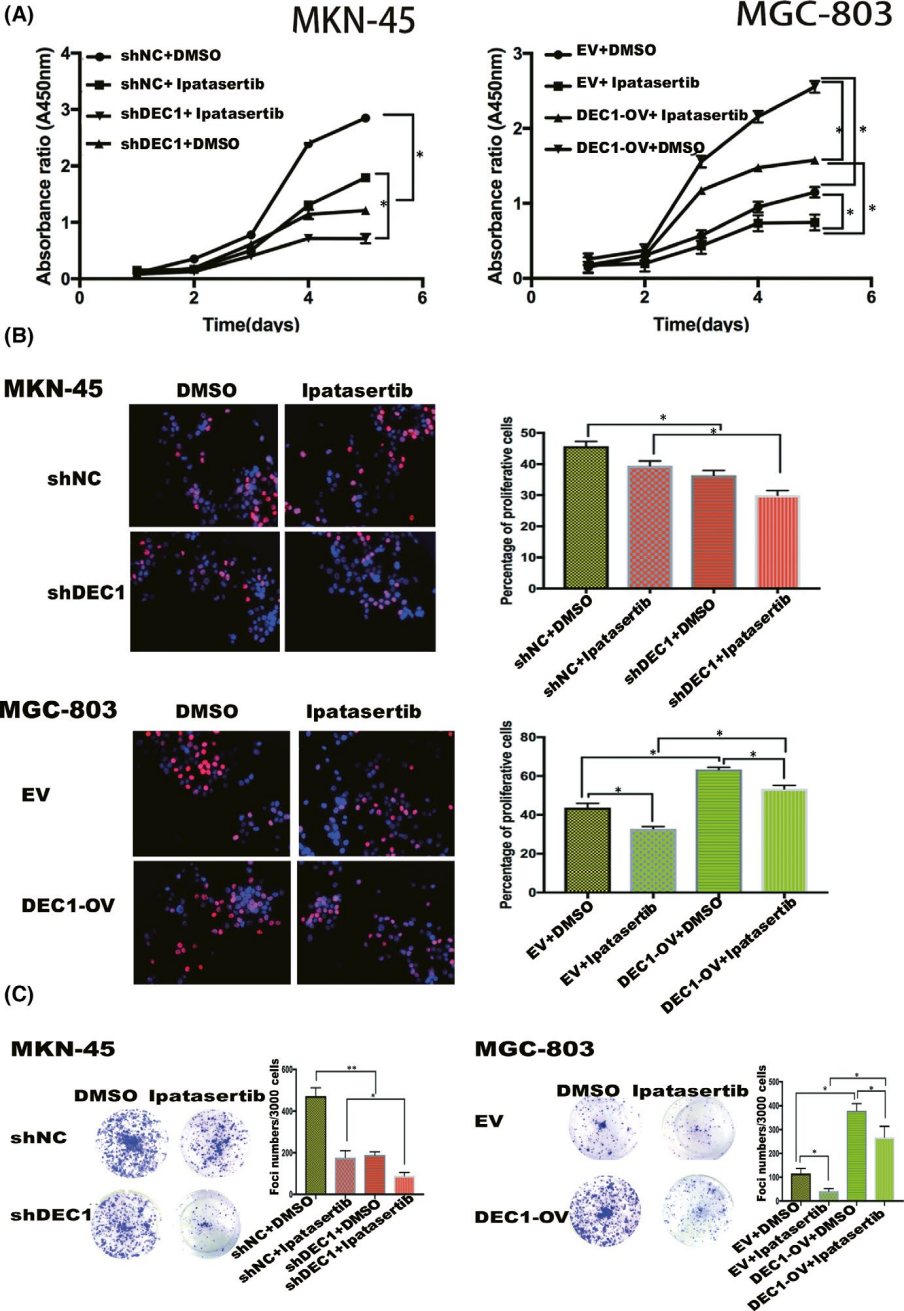
Akt/NF‐κB pathway is involved in DEC1‐mediated increased GC cells proliferation. A. The results of CCK‐8 showed the proliferation ability of MKN‐45‐shDEC1 and MGC‐803‐DEC1 overexpression after adding with Akt inhibitor ipatasertib. B‐C. The results of EdU experiment and cell clone formation experiment. EV, empty vector; OV, overexpression vector. *p < 0.05

To gain insights into the mechanisms by which DEC1 are linked to the progression of *H*. *pylori*‐positive GC, GSEA between low and high DEC1 expression was conducted based on the RNA‐seq data from GSE62254.^34^ *H*. *pylori*‐positive GC patients with higher DEC1 expression showed enrichment of genes associated with the epithelial cell signalling in Helicobacter pylori infecting, apoptosis and pathway in cancer (Figure 4A). Those pathways highlighted the association between DEC1 and *H*. *pylori*‐positive GC progression. Notably, Akt and NF‐κB were also significantly enriched in these pathways. The Akt/NF‐kB signalling pathway plays a role in the proliferation of tumour cells. We next tested whether *H*. *pylori* infection could activate Akt/NF‐kB pathway via enhancing DEC1 expression. As predicted, the Akt/NF‐κB pathway was activated in GC cells by *H*. *pylori* infection (Figure 4B). p‐Akt and NF‐κB expressions were inhibited by DEC1‐shRNA while increased by DEC1 overexpression (Figure 4C). In addition, ipatasertib, a specific inhibitor of Akt, was used in the present study to block the Akt/NF‐κB pathway (Figure 5). CCK‐8 results showed that ipatasertib further inhibited the proliferation of MKN‐45/DEC1‐sh cells. Ipatasertib partially offset the upregulated proliferation of MGC‐803/DEC1 cells (Figure 6A). The results of the EdU experiment and cell clone formation experiment were consistent with those of the CCK‐8 experiment (Figure 6B‐C).

**FIGURE 7:**
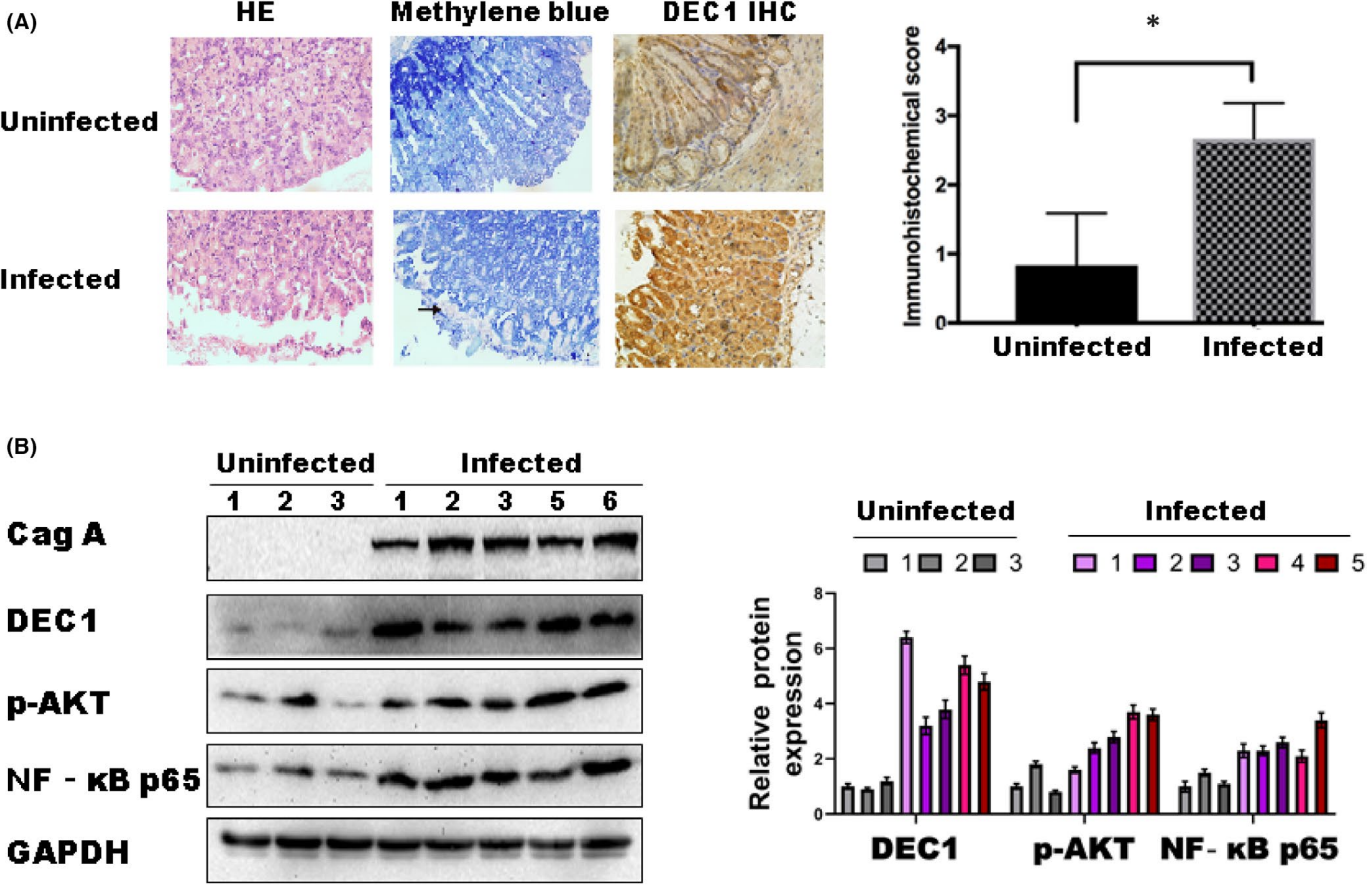
C57BL/6J mice got intragastric administration of standard *H*. *pylori* strain NCTC 11637. A. Pathological observation showed that chronic superficial gastritis was common in *H*. *pylori* gastric lavage group. Methylene blue staining (40 ×). *H*. *Pylori* infection and colonization were found in 20 of 25 mice in the gastric lavage group. Immunohistochemical analysis to detect DEC1 expression. B. Western blot analysis of total gastric mucosal protein in mice. *p < 0.05

We next performed in vivo studies to verify our results with a *H*. *pylori* infection mouse model. C57BL/6 mice were administrated with H. *pylori* 11673 to produce the mouse *H*. *pylori* infection model. The gastric mucosa was normal and displayed no inflammatory cell infiltration in the control group. The upregulation of DEC1 was markedly increased in *H*. *pylori* infectious mice compared with the control mice. Western blot results showed that the expression of p‐Akt and NF‐κB protein in *H*. *pylori*‐positive gastritis mice was significantly upregulated (Figure 7). Overall, our results suggest that DEC1 is critical to the activation of Akt/NF‐kB signalling pathway induced by *H*. *pylori* infection in GC.

## 4 DISCUSSION

In this study, we determined the pivotal role of DEC1 in uncontrolled proliferation and GC carcinogenesis induced by *H*. *pylori* infection. We observed that elevated DEC1 expression was directly correlated with GC *H*. *pylori*‐infected status and progression. We also identified the novel mechanistic link between the DEC1 expression and *H*. *pylori* infection in GC, through which *H*. *pylori* infection facilitates the proliferation of GC cells via the DEC1/Akt/ NF‐κB pathway.

DEC1 is involved in multiple biological and pathological processes including the development of cancer.^35^ Of note, DEC1 is induced by various stresses, and the increased expression of DEC1 is associated with tumour cell survival.^18^, ^19^, ^36^ An emerging view that DEC1‐mediated inflammatory responses can be beneficial in host defence against pathogens.^17^, ^37^ Our previous study has shown DEC1 upregulation promotes GC cell proliferation without the consideration of *H*. *pylori* infection (Jia, Hu et al. 2018). However, the expression status and roles of DEC1 that associated with *H*. *pylori* infection in the progression of GC remain elusive. Teng et al demonstrated that *H pylori* infection of gastric epithelial cells increases of DEC1 expression, which increases CD4+ T‐cell infiltration through promoting CXCL12 production.^38^ Their study focuses on the role of DEC1 in gastritis and the immune system. Only a small fraction of infected gastritis develops GC. Here, we describe the increased expression of DEC1 in *H*. *pylori*‐positive GC cells. Moreover, in GC patients, DEC1‐positive expression indicated a poorer prognosis and *H*. *pylori* infection leads to an increase of DEC1 expression, which can worsen the prognosis of GC. DEC1 is an attractive target for blocking the uncontrolled proliferation and gastric tumorigenesis induced by *H*. *pylori* infection. Bcl‐2, Bax and Survivin were regulated by *H*. *pylori* in GC cells, and the induction was reversed by DEC1 knockdown. These results indicated that DEC1 increased *H*. *pylori*‐induced motility in GC cells.

Given DEC1 tilts the balance towards inflammation versus disease transformation, it is important to determine the factors and signalling pathways that regulated by DEC1 in different contexts. We analysed the signal pathways correlated with DEC1 expression in *H*. *pylori*‐positive GC by using GEO database. Intriguingly, Helicobacter pylori infecting, apoptosis and pathway in cancer were positively associated with DEC1 expression. Indeed, our data demonstrate that *H*. *pylori*‐induced activation of Akt and NF‐κB were significantly upregulated by DEC1 expression in GC cells. In some previous reports on different cells, PI3K/Akt signalling pathway could regulate DEC1 expression.^39^, ^40^, ^41^ Our study found DEC1 appears to regulate Akt expression in GC cells, while Akt inhibitor has little effect on the DEC1 expression. This is an instance of complex cross‐regulation of DEC1 with another signalling pathway. Furthermore, there are functional link exists between the Akt and NF‐κB pathways. Akt signalling pathway actively regulates NF‐κB.^42^, ^43^ Therefore, we investigated the effect of Akt on NF‐κB through *H*. *pylori*‐regulated DEC1 in GC cells. We showed that regulation DEC1 and Akt expression could reverse *H*. *pylori*‐induced GC cells proliferation and NF‐κB activation. Consistent with the results from GC cell lines, DEC1, Akt and NF‐κB expression was increased in *H*. *pylori*‐infected gastritis tissues *in vivo*. It is plausible that activation of multiple cell proliferation pathways helps *H*. *pylori* to regulate the functions of multiple tumour suppressor genes and oncogenes, resulting in a higher proliferative capacity, more extensive spread and more rapid progression of *H*. *pylori*‐positive GC.

In conclusion, we define the important role of the DEC1 expression mediated by *H*. *pylori* infection in GC. We also found that, when induced by *H*. *pylori*, DEC1 overexpression targeted Akt/NF‐κB pathway, thereby leading to the promotion of cell proliferation. These results provide novel insights into the molecular mechanism of GC, with DEC1 serving as a potential therapeutic target for GC linked to *H*. *pylori*.

## CONFLICTS OF INTEREST

The authors declare no conflict of interest.

## AUTHOR CONTRIBUTIONS

**Yanfei Jia:** Conceptualization (equal); Data curation (equal); Formal analysis (equal); Funding acquisition (equal); Methodology (equal); Writing – original draft (equal). **Yanyan Liu:** Formal analysis (equal); Investigation (equal); Methodology (equal). **Jingyu Zhu:** Resources (lead); Validation (equal); Visualization (equal). **Liang Liu:** Investigation (equal); Resources (equal); Visualization (equal). **Xiaoli Ma:** Supervision (equal); Validation (equal); Visualization (equal). **Duanrui Liu:** Software (equal); Validation (equal). **Shuyi Han:** Resources (equal); Validation (equal). **Lulu Zhang:** Software (equal); Validation (equal); Visualization (equal). **Zhiqiang Ling:** Funding acquisition (equal); Project administration (equal); Supervision (equal). **Yunshan Wang:** Funding acquisition (lead); Project administration (lead).
